# Designing User-Centered Mobile Health Initiatives to Promote Healthy Behaviors for Children With Disabilities: Development and Usability Study

**DOI:** 10.2196/23877

**Published:** 2021-09-16

**Authors:** Keiko Shikako, Ebele R I Mogo, Valerie Grand-Maison, Robert Simpson, Lesley Pritchard-Wiart, Annette Majnemer, Jooay App Research Group

**Affiliations:** 1 School of Physical and Occupational Therapy McGill University Montreal, QC Canada; 2 Guelph University Montreal, QC Canada; 3 MAB-Mackay Centre School Montreal, QC Canada; 4 Department of Physical Therapy University of Alberta Edmonton, AB Canada; 5 McGill University Montreal, QC Canada

**Keywords:** implementation research, mobile health, children with disabilities, physical activity promotion, digital health, inclusive leisure participation, mobile phone

## Abstract

**Background:**

The gap between research and its practical application in community settings limits its impact on public health. Closing this gap has the potential to improve the well-being of underserved groups, such as children with disabilities. Mobile health has the potential to improve access to community resources and support for underserved populations, thereby encouraging improved health behaviors.

**Objective:**

In this feasibility pilot study, we describe the development of the mobile app Jooay. Jooay was developed in partnership with stakeholders to facilitate access to leisure and physical activity community programs for children and youth with disabilities. We also reflect on the lessons learned throughout the implementation process that are relevant for improving the health behaviors of children with disabilities.

**Methods:**

We used a participatory action research approach to develop the app. We also administered a survey to current Jooay users and analyzed various app usage indicators to explore use patterns, user feedback, and preferences. Finally, we critically appraised the implementation process through a best practices for implementation research framework.

**Results:**

We developed a product that responds to users’ identified need to find information and follows accessibility and user-centered design standards. The analysis of usage data revealed that access to the Jooay app is concentrated in urban areas. Perceptions, attitudes, and information needs varied according to the type of user. The use of the mobile app changed over time, and usage decreased after the app was downloaded, indicating a need for the sustained engagement of app users. Users found value in the ability to identify activities that they would not otherwise know about. However, app use alone was not sufficient to improve participation. Although the app was developed based on users’ active input in multiple iterations, we encountered challenges with survey recruitment and attrition, suggesting the need for more seamless and engaging means for collecting data within this population.

**Conclusions:**

Interactions between users and the app can sustain user engagement and behavior change. We will improve the app’s next iterations by using the information gained from this study to conduct a larger study to assess the relationship among social and material deprivation, urban design, and access to inclusive and adaptive leisure programs. This study will inform the improvement of app listings to improve the use of Jooay by different user groups and promote health through mobile apps for marginalized groups.

## Introduction

### Background

Implementation research involves studying research uptake and its effect on the outcomes of multiple stakeholders [1,2]. In medicine and public health, a significant gap remains between research knowledge and action, limiting the direct impact of research on health [1]. Closing this gap requires the consideration of multiple contextual variables, consideration of technology use, and other strategies to improve health behaviors [1].

Innovative strategies for facilitating changes in health behavior are particularly important for underserved communities and populations. People with disabilities comprise approximately 15% of the global population and are at risk of poor health outcomes [3]. Children and youth with disabilities face challenges in accessing health services and health-promoting activities [4]. They rarely meet the recommended standards for physical activity and have lower physical activity levels than their peers [5-8]. They also have a higher prevalence of noncommunicable diseases, such as obesity, diabetes mellitus, and coronary artery disease [8], and are disproportionately affected by environmental, socioeconomic, and interpersonal barriers to healthy lifestyles [8]. Although parents or other caregivers (hereafter caregivers) value their children’s participation in physical activities, they face multiple participation challenges, such as inadequate access to adapted programs and inclusive settings [9-11].

Health promotion initiatives can improve outcomes for children living with a diverse range of social, emotional, and behavioral disabilities [12-14]. Contextual factors that can serve as participation barriers or facilitators for children with disabilities include information about activities, the cost of activities, the accessibility of facilities, and the presence of trained staff and support [3,15-19]. The alignment of needs of people with disabilities with effective health promotion initiatives can foster better health outcomes, provide a sense of empowerment, reduce health disparities, and improve overall individual quality of life and community well-being [15].

Mobile health (mHealth) is gaining primacy for the creation of targeted, accessible, and context-appropriate health promotion solutions. mHealth tools include various devices, software, and solutions that use mobile phones to improve health [20]. Potential benefits include time savings, convenience, and improved access to underserved populations [21-23]. mHealth tools have also improved health behaviors among young people and in chronic disease management [14,24,25].

Although there have been several pilot studies on mHealth interventions, knowledge gaps remain regarding the appropriate development and use of mHealth to promote health equitably [22]. Preliminary studies suggest that users of mHealth are younger, more educated, have better health, and belong to a higher income group than nonusers [26]. Despite its promise in improving health, mHealth may actually exacerbate health disparities if underserved populations do not have access to digital tools and the ability to develop literacy in using them.

In addition, research on mHealth has not yet sufficiently demonstrated efficacy, effectiveness, user engagement, effective scale-up, and competitive value [21,27]. Many mHealth efforts have been inadequately designed for implementation and evaluation [21]. Evaluations that do exist do not prioritize data disaggregation, limiting considerations of equity and impact for marginalized groups [21,22].

### Objective

In this study, we explore the feasibility of mHealth to improve access to information on community-based inclusive leisure activities for children with disabilities. Our specific objectives are to (1) describe the development process of the mHealth solution and (2) identify use patterns and user preferences. The secondary objective is to establish the feasibility of using a mobile app to test behavior change and to pilot test data collection through app analytics and users.

Understanding the development, implementation, and uptake of mHealth can facilitate the design and use of future technologies for health promotion in high-risk groups. The knowledge gained will also inform the scale-up of the app and, more broadly, the field of implementation research.

## Methods

### Overview

In this pilot feasibility study [28], we asked the following question: can a mobile app be developed in collaboration with stakeholders and used to promote health behavior changes in children with disabilities, and if so, how? (feasibility component). We also conducted a pilot study to assess the extent to which the app supports health behavior change (pilot component). In this paper, we describe the app development process and the results of a small-scale survey with a subset of app users.

We adopted a hybrid implementation research design [29], whereby the intervention or solution is developed and tested concurrently instead of using the traditional approach, in which development is conducted before the intervention is tested with the population comes first and then is tested out in the population followed by testing. The value of hybrid designs resides in the possibility of cocreating knowledge while simultaneously incorporating and testing intervention improvements. Multiple iterations required in a technology development project make the hybrid design an optimal approach to effectively test and implement user-responsive mHealth-based interventions.

Ethical approval for this study was obtained from the McGill University Institutional Ethics Review Board as well as the Centre for Interdisciplinary Research in Rehabilitation.

### Theoretical Frameworks

**Figure 1:**
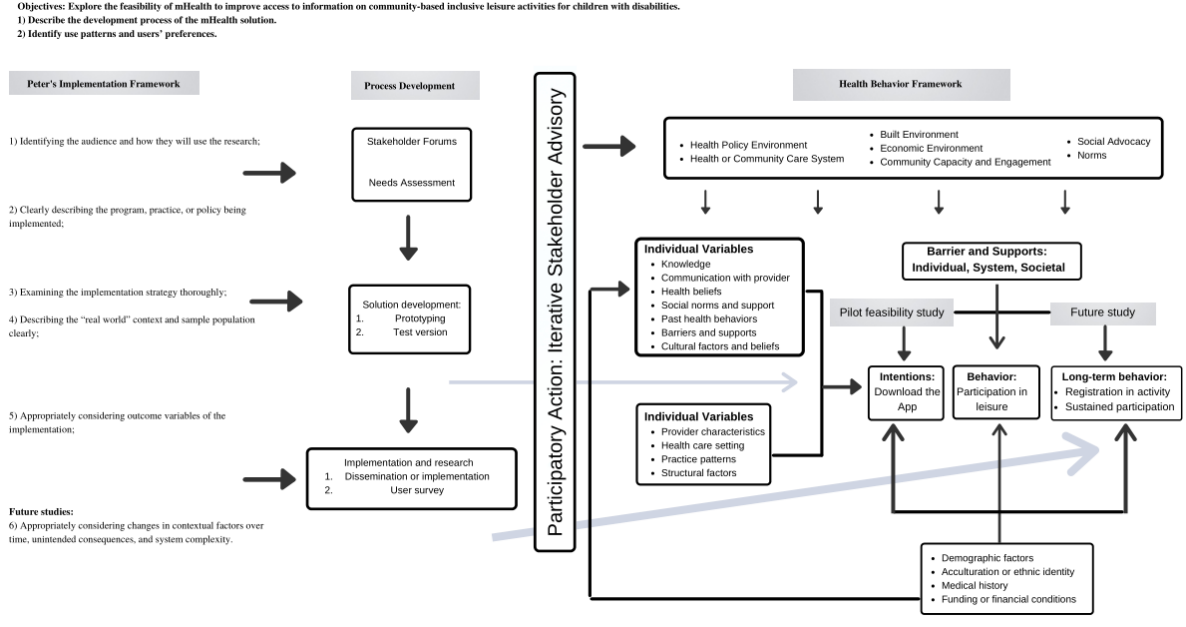
Integration of theoretical frameworks. mHealth: mobile health.

Several theoretical frameworks informed our approach and study objectives. Figure 1 illustrates the integration of these frameworks with the project objectives and procedures.

The overall approach to app development and evaluation was based on the participatory action research (PAR) framework [30]. PAR aims to share power between researchers and those being researched. Our approach was thus reflective and iterative and involved multiple stakeholders throughout the research process, from the elaboration of the initial research question to app development and evaluation.

The overarching objective and the development process (specific objective 1) were informed by the best practices for implementation research prescribed by Peters et al [31]. Specifically, we used the following steps: (1) identify the audience and how they will use the research; (2) clearly describe the program, practice, or policy being implemented; (3) examine the implementation strategy thoroughly; (4) describe the real-world context and sample population clearly; and (5) appropriately consider outcome variables of the implementation. The last 2 steps consider specific context variables and appropriately consider changes in contextual factors over time, unintended consequences, and system complexity, which will be considered in future studies.

To expand on step 5 (appropriately considering outcome variables of the implementation), we adopted the Health Behavior Framework [32]. This framework includes a detailed consideration of outcome variables related to the expected implementation (ie, use of mHealth technology, the Jooay app). The elements of the framework informed the key aspects to consider while identifying users’ patterns and preferences (specific objective 2). Determination of the feasibility of use requires a comprehensive understanding of the variables involved in the health behavior change proposed by this framework.

### mHealth Tool Development

#### Overview

Our pilot intervention tool is Jooay, a free mobile app that aggregates information on leisure activities for children and youth with disabilities aged 6-21 years in Canada. The Jooay app was launched in the spring of 2015 on iOS and other web platforms. The version used for this pilot study listed approximately 1000 activities distributed in 5 of the 10 Canadian provinces.

#### Stakeholder Forums

First, we organized 4 stakeholder forums across the Canadian provinces. Participants were a purposeful sample of youth with disabilities, caregivers of children with disabilities, health and education providers, policy makers, and community organization leaders. They were invited by pediatric rehabilitation center collaborators and city leisure departments in Montreal, Toronto, Calgary, and Vancouver.

Each forum was a 1-day event with the following objectives: (1) to present the current research evidence on determinants of leisure participation for children and youth with disabilities and (2) to identify strategies to improve access to leisure opportunities for children and youth with disabilities across Canada.

Using a business canvas model [33], participants were grouped according to the stakeholder group to which they belonged (health care providers, caregivers, youth with disabilities, policy makers, and grassroots organizations) and were asked to consider barriers to access to leisure participation. Subsequently, they were grouped into mixed groups representing different stakeholders to discuss implementation solutions to promote the participation of children with disabilities.

Each participant was invited to write their selected *top solution* on a card. Instructions were that the *top solution* depicted in the card should be actionable, be feasible, and have an impact on the participation levels of children with disabilities. Participants then engaged in a prioritization exercise in which they exchanged cards and, in pairs, compared and ranked 2 ideas. The solution that ranked higher at the end of 5 rounds of paired ranking was considered the one that, according to the forum participants, would yield the highest impact on participation in leisure for children with disabilities. The solution ranked as the most feasible and promising solution to overcome systems barriers and promote participation in leisure across the 4 forums was “[a]n electronic list of inclusive and adapted activities.” Participants in each forum volunteered to form a working group to support the development of the idea.

#### Advisory Group

The Jooay app was developed in 2 phases: (1) prototype development and (2) test version development.

A convenience sample of 5 users (2 caregivers, 1 physical educator, and 2 occupational therapists [OTs]) and several research team members (including community organization leaders and physical and recreational therapists) provided input into the user interface development, user experience development, and final test versions during both phases.

A larger group of stakeholders comprising clinicians (OTs and physical therapists), physical educators, caregivers of children with disabilities, and representatives of community organizations constituted the user partners who provided input during the development phase of the app. These stakeholders provided insights into the best features and types of information to be listed and were involved in the conception of the app, the testing of multiple versions, and the development of the research protocols to address the feasibility and pilot testing of the app. The stakeholders were chosen based on the following considerations: (1) willingness to participate; (2) direct experience working or advocating for children with disabilities; and (3) representation across diverse areas of involvement that included pediatric rehabilitation centers, school boards, community organizations, newsletters of organizations, and networks related to adapted leisure and addressing caregivers of children with disabilities.

A dynamic protocol for testing and responses was established using a collaboration platform and the research team made connections between the developers and user testers.

#### Prototyping and Test Versions Development

##### User Experience

The mobile app was initially developed as a prototype during a hackathon (weekend event grouping developers, user experience designers, programmers, and project managers). The first test version was developed for iOS, Android, and the web. It was made available to users free of charge through regular channels (App Store, Google Play, and website).

##### Development

A partnership with an external mobile app developer was necessary to secure further development, and multiple funding sources were required to attain industry standards. Suggestions that arose from user partners and technology developers resulted in the creation of 3 native platforms for iOS, Android, and the web.

Core technology developments included mandatory implementation of full accessibility protocols for mobile and web platforms (including voice-over, voice control, color contrast, and easy access—a feature available in the iOS and Android accessibility protocols where buttons and number of clicks to action are reduced). An accessibility consultant was hired to test and assess these features during development.

##### Content

The participants provided important information on preferences for the type of information displayed for each activity listed. The selection of included domains had an impact on development cost; therefore, there was an assessment of the most relevant information components to be retained in the app.

The final domains reflected stakeholders’ preferences for information and included activity description, types of equipment required, type of disability (eg, physical, intellectual, or those classified as *all are welcome*), cost, and time frame. The same decision algorithm was used to create filters within the map search as well as to collect basic demographic information from users when they registered to use the app.

Stakeholders also suggested a list of other resources to be listed on Jooay, such as reference links to other types of supports toward leisure participation, such as respite care and support groups for youth and caregivers, research related to leisure, and the Jooay web-based Facebook community. Our user partners supported the development of the survey questions by reviewing the initial questions and providing feedback on how to phrase the questions, question content, and structure (eg, options for participants to suggest other fields or useful resources that should be added to the app). They also supported survey distribution to a larger sample of community organizations and rehabilitation centers, in addition to the list of app users, and acted as champions to disseminate the survey and the app as a product to other users.

Activities in the app were initially populated on the basis of pre-existing lists of adapted and inclusive leisure activities from pediatric rehabilitation centers in the targeted provinces and schools serving children with disabilities in 6 provincial capitals across Canada. Using the key terms identified on the websites of these organizations, the research team searched for additional activities and continued populating the app database.

### Data Collection and Sample

This pilot project used 2 sources of data: (1) analytics of the Jooay app users and (2) electronic surveys sent to registered participants.

#### Participants

Through a preliminary analysis of app analytics data from approximately 600 users, we determined that approximately half were health or education service providers and the other half were caregivers of children with disabilities. No further sociodemographic information was required from the users upon registration. Furthermore, 2 different surveys were subsequently developed to target service providers and caregivers. This pilot study was conducted with all app users who had registered with the app using their email. Registration was not a mandatory requirement to gain access to information on Jooay.

A total of 273 of approximately 600 user emails were initially available. Additional users who downloaded the app and registered during the 6-month data collection period also received an invitation to participate. A brief explanation of the study and survey links were also posted through social media channels, specifically through parent support groups and the Jooay page on Facebook, asking app users who might not have registered emails to complete the survey. Study knowledge brokers in pediatric rehabilitation centers, school boards, community organizations, newsletters of organizations, and networks related to adapted leisure and addressing caregivers of children with disabilities also shared information about the app and survey. Additional participants in the regions where the activities were published on Jooay at the time of the study, namely, the 6 provinces—Alberta, British Columbia, Quebec, Nova Scotia, Saskatchewan, and Ontario—were targeted in addition to existing users of the app.

Participants were required to own a smartphone, have access to the internet, and understand English or French. Users aged <13 years were excluded from the survey, given that 13 was the established minimum age to own social media accounts, such as Facebook and Twitter, and the legal age required to download Jooay from the App Store. Assent was required for participants aged <18 years.

#### Procedures

Potential participants received an email with a brief explanation of the study and a link to a web-based survey on REDCap (Research Electronic Data Capture; developed by Vanderbilt University) hosted in a research database. Participants were prompted to provide consent before the initiation of the survey. A total of 5 invitations were sent by email within a 2-week interval.

Confidentiality and anonymity of survey responses were ensured by deidentifying survey responses. Participant emails were only used to prompt their participation in the study and were not associated with their answers. A 2-step password-protected REDCap account accessible only to the survey team was used to ensure data privacy. The survey questions analyzed as part of this pilot study are provided in Textbox 1.

**Survey questions analyzed as part of this study.**

**Questions about the app and app use**

1. Rank the relevance of the current existing features of the app (are these sections important in helping you find an activity to pursue?)

2. Was information in the following sections helpful in finding activities?

- cost
- type of activity
- description of activity
- type of disability
- location
- age range
- schedule
- season
- reviews or ratings
- other links and related information

**Questions with "agree," "neutral," or "disagree" responses**

1. The app is easy to use

2. The app has a comprehensive list of existing activities

3. The information on the app is accurate

**How did you learn about Jooay?**

1. Social media

2. Health or education professional

3. Other sources (which one?)

**Open-ended questions**

1. What feature you would like to see in the app that is currently not available?

2. What are main issues of the app?

3. Do you intend to uninstall the app? (yes or no)

If yes, explain why (open ended)

**Questions about health behavior**

1. The person for whom the app information is being used (child, client, or student) has engaged in regular leisure activities before using the app? (yes or no)

2. My child, client, or student participation in leisure has increased because of information

found in the app? (not at all, a little-moderately, or a lot)

If participation improved, explain why (open ended)

3. The activities in the app fits my, my child’s, or my client’s needs (yes—moderately—no)

**Sociodemographic questions**

1. Your age (respondent)

2. Age of the person with disabilities for whom you’re using the app info

3. Province of residence

### Analysis

After the REDCap surveys were distributed [34], responses were exported into SPSS (IBM Inc), and responses from the English and French surveys were merged. Descriptive analyses were conducted for participant characteristics, app usage patterns, and preferences for app features and information. We also explored the associations between the sociodemographic characteristics of caregivers (age and gender), with perceptions of app features, app use patterns, and other characteristics.

The question of power calculation is often a challenge in assessing the impact of mHealth tools on health outcomes [35], given the challenges of generating a significant sample size and reducing attrition. This pilot study also aimed to set parameters for sample size calculations for future research using this mHealth tool.

## Results

The results of the development process and feasibility testing are presented in the context of the 2 guiding theoretical frameworks: the Implementation Framework and the Health Behavior Framework. We identify which domains of these frameworks are addressed in each of the following sections.

### Development Process of the mHealth Solution

*Implementation Framework: Identifying the audience and how they will use the research*

*Health Behavior Framework: Health policy environment, health or community care system, community capacity and engagement, and social advocacy*

More than 200 stakeholders representing diverse groups participated in the development process of the mHealth solution. An average of 50 participants in each of the 4 stakeholder forums identified that a mobile listing of leisure activities was the most desirable and feasible solution to promote participation in leisure activities for children with disabilities.

**Figure 2:**
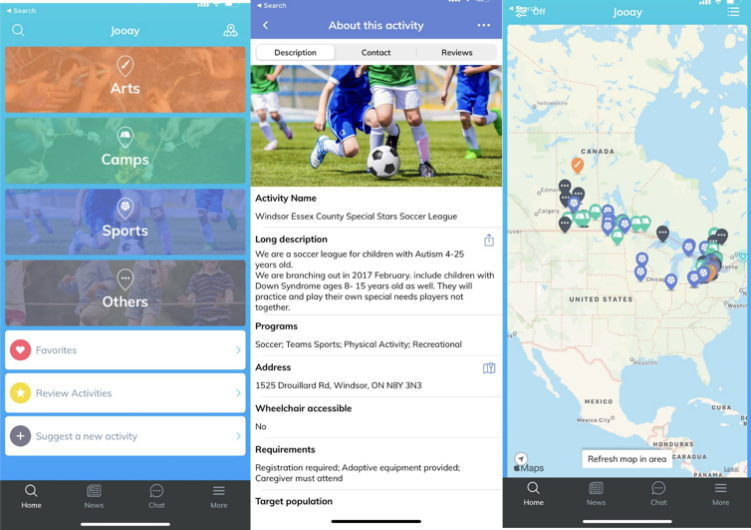
Screenshots of the final product (Jooay App).

Table 1 describes the main steps of development, the strategies adopted, and the main outputs in each step. Figure 2 shows screenshots of the final app that was developed.

**Table 1:** Development process and outputs.

Steps of development	Strategy	Output
Needs assessment	Stakeholder forum	Mobile app with dynamic and interactive list of leisure activities in the community, based on geolocation (close to where children live)
Design and prototyping	Hackathon	Branding to represent multiple disability groups; English and French languages
User interface	Stakeholder advisory	Accessibility features beyond basic protocols Minimum information requirements upon registration Additional information asked for research from users (eg, sociodemographics) Additional information given to users (eg, research about leisure and respite care) Domains
User experience	Stakeholder advisory	Accessibility features include visual impairment, cognitive impairment, testing of map functions, and multiple platform accessibility features (iOS, Android, and web) Easy access to key information by different users: parents versus service providers
Test versions	Stakeholder advisory Collaboration platform (Trello; developed by Atlassian) Third part development company	Sustainable ways to provide feedback from users to developers (email)
Pilot version	Public at large	Need to create community among users (eg, chat or group interactions) Sustainability: maintenance of updated information is crucial; maintenance of technology in each of the native platforms (cost) Crowdsourcing: make possible for organizations and users to suggest activities Troubleshooting: need for ongoing technology support to maintain the app relevant users’ satisfaction-expected health outcomes

Stakeholders in the forums represented a range of health and policy environments, communities, and health care systems. They presented the key factors for the use of the app in the health and community environments (eg, information that should be added to make the mHealth solution relevant). Stakeholders in the forums and in the advisory group also contributed the listings that they currently had in the municipal, local listings of activities to the database and engaged in social advocacy.

### User Patterns and Preferences

*Implementation Framework: Examining the implementation strategy thoroughly, describing the sample population clearly*

*Health Behavior Framework individual variables: knowledge, health beliefs, social norms and supports, cultural factors and beliefs, barriers and supports, and structural factors*

A total of 239 participants received the survey. The response rate was 38.9% (93/239). Table 2 includes participant characteristics. Twenty-four participants responded to the question about age of the person for whom they were seeking activities. From those 62% (15/24) of participants indicated that they were seeking information for children aged 4-12 years.

**Table 2:** Survey participants’ characteristics (N=93).

Sample characteristics			Participant, n (%)
Caregivers of children and youth with disabilities			38 (41)
Health care or education service providers			20 (22)
Youth with disabilities			4 (4)
Other stakeholder groups			31 (67)
**Living in urban area**			
	Caregivers	31 (82)	
	Service providers	18 (92)	
**Gender (female)**			
	Caregivers	34 (89)	
	Service providers	15 (76)	

**Figure 3:**
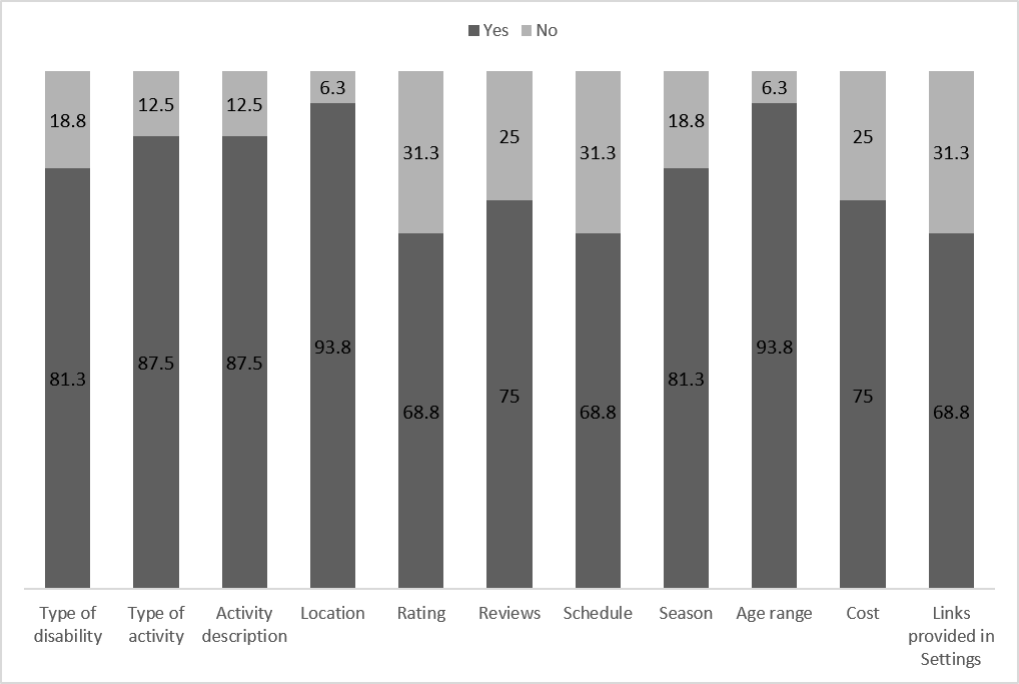
Perception of users about the helpfulness of app sections.

Participants were asked to rank the relevance of app features. Participants reported that the most useful information was age range and location, followed by the activity type and description of activity. Most participants found that information in all sections of the app was helpful; sections that had less information, such as reviews and ratings (which is expected because the app is new and not many reviews had been done yet), were perceived as less helpful (Figure 3). The links provided in the settings sections included research summaries and were among the least useful sections, along with activity schedule, probably due to the frequent change in schedules, making the information not accurate.

Participants were asked to indicate (open-ended questions) the features they would like to see added to the app. A total of 34 participants responded to this question, and their responses were categorized as follows: development of a web-based community or forum (13/34, 38%), a means to track their participation in leisure and physical activities (8/34, 23%), positive prompts for action (7/34, 20%), and a points or rewards system to incentivize participation (6/34, 18%).

When asked about their concerns with the app, the main negative feedback was related to the insufficiency of listings (9/26, 35%), followed by the lack of activities in the regions where participants worked or lived (4/26, 15%). Half of the respondents agreed that the app was easy to use and accessible. However, 37% (10/26) disagreed that it was comprehensive, and 62.5% (16/26) indicated that they were neutral about whether the information was accurate. Although 85% (17/20) of the service providers using the app noted that they did not intend to uninstall the app, 72% (27/38) of the caregivers said they might intend to.

We asked participants to identify how they heard about the Jooay app as a means of increasing our understanding of how information spreads in this population. Overall, 22% (20/93) of respondents indicated social media as their source of information, followed by those who indicated that they heard about it from their physical therapist, OT, or recreational therapist (18/93, 20%); 32% (30/93) indicated *other* sources, such as word of mouth or through advertising in hospitals.

Respondents had the opportunity to respond to open-ended questions on whether the app contributed to increased child leisure participation, and if so, how. One of the ways in which participants indicated that the app contributed to increased participation in leisure was by raising their awareness of community resources and programs. Service providers (clinicians and educators) who responded to the survey noted that they had used the app to provide information to families, caregivers, and clients about leisure opportunities, but they could not indicate whether the app had contributed to an actual increase in leisure participation. Caregivers indicated that although the app increased their awareness of activities that they were not aware of, it was still challenging to find activities that were suitable for their children. They indicated that the app needed to include more activities to enhance opportunities for participation.

### Associations

We originally intended to explore the associations of sociodemographic characteristics, such as age, gender, type of disability, and place of residence, with perceptions of the app’s features and app use patterns. However, a significant amount of missing data (61%-91% of data are missing for some variables) precluded us from doing so. However, the preliminary exploratory analysis identified significant associations between the age of the caregiver and whether the person with a disability had engaged in physical activity before using the Jooay app (n=16; Fisher-Freeman-Halton Exact test: *P*=.01). Most of the caregivers indicated that their child was actively engaging in physical activities before accessing the Jooay app (28/38, 75%).

There was also a significant relationship among the users who indicated that the app had the information they were looking for and the province where they were located (n=20; Fisher-Freeman-Halton Exact test: *P*=.05). Most of the responses (12/20, 60%) were from Quebec, and of those responses, 9 (75%) respondents found that the app had a moderate fit with their needs. The lack of diversity in the users’ responses precluded a valid test of the association between user type (caregiver vs professional) and whether users found the activities in the app were a good fit; however, half of the respondents in both categories (n=10) indicated that the activities they found in the app had moderate to no fit with what they were looking for.

There was insufficient data from multiple user types to test for an association between user type and whether participation of their child increased as a result of the use of the Jooay app. Most of the respondents to this category of questions were caregivers (19/20, 94%), and 84% (17/20) of them indicated that their child’s participation increased after app use only slightly or moderately.

There was also an association between user type (caregiver vs service provider) and how frequently they used the app (n=24; Fisher-Freeman-Halton Exact test: *P*=.005). Most of the respondents to this category of questions were community organizations (7/24, 29%) and health care professionals (9/24, 37%); 45% (11/24) of respondents reported using the app multiple times without finding an appropriate activity for their client or child. The other 55% (13/24) of respondents had used the app less frequently and did not indicate whether they found an activity they were looking for or were only browsing through activities or exploring the app.

## Discussion

### Principal Findings

Using a PAR approach, we codeveloped and pilot-tested an mHealth tool with stakeholders to improve access to information and participation in inclusive leisure activities for Canadian children and youth with disabilities. Our aims were to understand feasibility aspects related to the use of a stakeholder-driven mHealth solution and understand user preferences and patterns to inform efforts to improve participation in leisure for children and youth with disabilities.

We used the Health Behavior Framework [32] to explore the individual and social characteristics of users and their relationship with the intention to use the app, measured as downloading the app, as well as the relationship with actual behavior change, reflected in increased participation. This enabled us to explore the potential impact of the app through users’ perceptions of usefulness, preferences for specific content, and suggestions for modifications. We also began to explore the sociodemographic characteristics of users and how they relate with use of mHealth technology and the expected health behavior outcomes.

We applied the implementation framework by Peters et al [31] to understand the process-based factors that shaped the development and use of the app and opportunities to improve them. Below, we discuss the lessons learned, challenges faced, and implications for larger-scale efforts to use mHealth technology to promote health for children and youth with disabilities.

### Process Development Challenges and Opportunities

Participatory research is valuable and has potential key outcomes in health and implementation science [36]. Important challenges to consider in co-designing technology with multiple stakeholders include the ethical challenges of developing study protocols that are constantly changing and require multiple ethics review board amendments, the need to respond to divergent opinions in all steps of a project (eg, questionnaires and administration forms) and product development (eg, user interface vs user experiments design phases), and the extended length of time necessary for an authentic co-design process. Most of these issues have been identified in previous reports on participatory research; however, additional challenges learned in this study include communication barriers between end user stakeholders and the technology development team because of divergent language, culture, and operational modes.

### Ethics

We maintained a close discussion about the nature of the project with the institutional ethics review board and agreed on the elements that did not require ethics approval (ie, the stakeholder forums and app development stages) and which elements did (the surveys sent to app users and the information taken from app analytics). For elements requiring ethics approval, we agreed to an open protocol with the core elements of the project being initially approved through the regular, extended ethics review board procedures, and future iterations (ie, length of activity description, recruitment materials, or wording changes requested by our user collaborators) to undergo an expedited review, allowing for a reduced turnover time.

### Stakeholder Engagement and Co-Design

It is important to advance implementation science on mHealth; it requires careful consideration of the interaction between technology development, participatory research and stakeholders involvement. We must develop protocols and standard operational procedures detailing aspects such as legal agreements between industry and research partners and business development plans that include design and maintenance discussions, establish a clear communication platform and verify with end users that they are able to access it. Flexibility in accepting other forms of communication that may be preferred by stakeholders have to be considered, and detailed note taking, with designated communication contacts on the team, is ideal.

### Technology Development, User Preferences, and Health Behavior Change

The vast majority of the app users found that the information contained in the app was relevant, albeit not comprehensive (ie, not enough activities listed in their region of residence), to really affect the desired health behavior change of increased participation for their child. This may have been the reason why 72% of caregivers planned to uninstall the app. Service providers indicated that they did not intend to uninstall the app; thus, they likely perceived greater utility. Several studies have shown that lack of information is one of the key barriers to participation and perception of good health services for families of children and youth with disabilities [37-39]. Creating a mobile app that has accurate, up-to-date information and responds to the users’ needs and preferences is a challenge but is also essential to health behavior change.

The lack of comprehensive information about activities in the app can be attributed to 2 main reasons: (1) the scarcity of activities offered in the community for this population and (2) the limited capacity to generate a comprehensive list of existing activities manually. The first issue is being addressed in a separate study (E Mogo, K Shikako, and A Majnemer; unpublished data; June 2021) where we conducted an in-depth analysis of the sociodemographic characteristics of regions and the availability of inclusive leisure activities, as listed in the app, with the objective of informing policy and program creation.

The second challenge relates to technology development. Creating comprehensive listings of activities that are constantly changing is a key challenge that can be addressed through technology but requires extensive sustainability planning. Sustainability and business models of mobile technology may be typical in design and industry but are foreign in health research when primary funds for development and co-design are obtained through research grants of limited duration. A sustainability model for mHealth needs to be further developed and tested to ensure the efficient use of research and user resources [40-42].

### Population Characteristics and mHealth Characteristics

Most survey respondents were caregivers of children with disabilities living in urban areas. Most activities listed in the app are located in urban areas. In the stakeholder forums, participants indicated that it is paramount to consider mHealth solutions that target populations that face multiple layers of marginalization, such as those who live in rural areas, indigenous children and families, and those who may not have access to mobile technology. Caregivers and service providers in rural areas have been identified as populations lacking access to services and other resources [43,44]. Therefore, the utility of Jooay as an mHealth tool in these regions is limited. We intend to apply these data to inform policy on gaps in service provision.

This pilot study sheds light on the challenges of including proxies as the main users of interventions. The target population using the app are caregivers and service providers, but the actual expected behavior change (participation in leisure) is targeted at the children under their care. Information on the participation patterns of families came mainly from caregivers providing answers to the survey. We identified an association between previous participation levels and the caregivers’ age and between their familiarity with apps and the technology associated with the actual frequency of use of the Jooay app. It is known that caregiver behavior regarding leisure has an influence on the child’s level of participation [45]. The primary respondents of the survey were female (83/93, 89%), indicating that health promotion efforts could target female caregivers to affect the health behaviors of children with disabilities.

Another important characteristic of this particular mHealth solution was accessibility for persons with disabilities. Although the app users are not necessarily children and youth with disabilities (only 8/93, 9% of our sample were persons with disabilities themselves) but rather the caregivers, it is important to consider that a mobile app for persons with disabilities should comply with accessibility standards. The challenges of following accessibility standards were perceived by our accessibility consultant and were outlined in previous research [46]. It was clear from the multiple iterations of testing that industry accessibility standards are not fully accessible for different individual needs, a factor that will be considered in future app development and iterations.

### mHealth and Health Behavior Outcomes

The survey results suggested that participants were not sure of the impact of the app on their children or clients’ participation levels but that they were certain that their knowledge about existing community activities had increased because of the app use. Ideally, mHealth should include artificial intelligence to directly track participation and objectively quantify the increase in participation as the desired outcome [47]. Although missing data prevented measuring desired behavior changes, a side effect of app use noted by some participants was the building community. Although the use of web-based communities by caregivers of patients with chronic health conditions is a relatively new phenomenon, several benefits and challenges have been identified [48]. Perceived benefits include connecting with others with similar lived experiences and challenges and increasing awareness about a medical condition or, in the present case, about existing activities and resources. Participants in this study indicated that they may be using the app not just as a resource to change health behaviors but also as a resource to connect to others and increase their awareness about possible activities, even if they are not available in the region where they live. Such indirect positive outcomes are worth investigating further. Public health implementation efforts should consider the power of connecting people and the possibilities brought about by mHealth technologies on this front.

For effective mHealth implementation, movement beyond pilot studies is needed to better understand the characteristics, preferences, and real-time use patterns of users. Partnership development with community organizations, cities, and other layers of governments is also needed to identify solutions to link resource databases and create machine learning algorithms that maintain relevant information for the public.

### Limitations and Future Directions

This pilot feasibility study faced limitations that supported several important considerations for future studies. First, this study was conducted in a real-world setting. We had no control over the location, type of activity offered, and the match between these activities and the participants’ preferences or needs. Previous research has indicated that preference for certain types of activities is associated with engagement in these activities [49]. This pilot study shows that, in fact, preferences are not easily matched to the availability of resources in a community, and this is a barrier to participation. This study sheds light on the importance of adapting individualized mHealth interventions to public health impacts.

The implementation strategy for this project was built in partnership with stakeholders. Implementation strategies included word of mouth and the use of local and web-based champions to disseminate information about the app and invite people to download it and use it. This project informed the important aspects of the implementation strategy on a larger scale. Recent studies have assessed the implementation of different data collection strategies through mobile apps and have reported mixed results. One study found that improving compliance with medication through digital data entry was feasible and reliable in a population of adults with HIV/AIDS [50]. Alternatively, another study that compared 3 electronic data collection methods for patients with a urinary tract infection—mobile app, electronic survey, or text message [51]—found no differences in response rates. They concluded that participants often stopped data completion after their first interaction with technology, leading to missing data. They also raise the issue of the variability of user demographics as a factor influencing response rates and preferences. We found similar challenges in completion rates. Our survey had a low response rate of approximately 39.83% and missing data, which limited the ability to make generalizations. Our stakeholder advisory group confirmed that individuals are highly interested in using the app but will respond only to very short surveys. We noticed through backend data that people often stopped completing the survey at the point at which they had to scroll through and sign the consent form before completing the survey, which also suggests the need to review ethics procedures when using mHealth technologies to conduct research.

Improvements in the recruitment strategy that will be implemented in the next phase of this study are the use of push notifications directly through the app, the shortening of the consent form to the minimal requirements, and the shortening of the survey. We will also ask our parent-partners, clinicians, and other coinvestigators to design a message that will be sent through push notifications. This message should be more welcoming of the participants’ responses. Future implementation efforts include making registration mandatory to use the app, increasing the relevance of information by implementing machine learning to update information, and increasing opportunities for interactions (ie, through push notifications and gamification). Future research must also address potential ethical constraints by appropriately adjusting their study design to elicit participation by using a mobile app as the only source for data collection.

The limited capacity of using app analytics across platforms and automated database updates imposed limitations to the data collected. Artificial intelligence to directly track participation, to objectively quantify increases in participation as the desired outcome, and improved analytics protocols are necessary to evaluate the effects on health behavior [47]. Therefore, monetary commitments associated with these strategies must be considered. Indeed, a thorough cost-effectiveness analysis should be an integral part of the scale-up implementation efforts of any technology [52].

Large-scale implementation research efforts using mHealth will need to consider better ways to engage the ecosystem of stakeholders, from users to rehabilitation centers, and community-based leisure centers to scale up and test more complex interventions. Efforts to elicit information from this population may require automated modes for data collection and partnerships between municipalities and organizations to link databases of activities and the app.

### Conclusions

Implementation research holds the potential to drive real increments in public health by translating research findings to real-world testing. At-risk and underserved populations, such as people with disabilities, require increased efforts toward change, as they face multiple contextual barriers often leading to poor health outcomes [3].

This study piloted the development and use of Jooay, a mobile app listing inclusive leisure activities. We also sought to understand user demographics and characteristics and the corresponding variables that would be of value to support its scale-up and effectiveness testing. Our intended use of this information was to better meet the needs of children and young people with disabilities and their support systems while also informing the literature to guide similar efforts.

mHealth is promising as a viable and feasible tool to execute implementation efforts, especially for this subpopulation. mHealth tools should integrate health promotion strategies for children with disabilities considering how to overcome poverty [3], be enjoyable [19], improve access to care [15], sensitize their health care providers, be person-centered, and provide the needed support for them to engage in healthy lifestyles [3,10,15,17,18]. These tools will also have to be supported by more information to support their efficacy, effectiveness, cost utility, and engagement. Scale-up studies are necessary to move mHealth development science beyond pilot studies [21,27]. Finally, information on the demographics and characteristics of mHealth users and the impact of mHealth on behavioral predictors and health behaviors is needed [26]. This can happen if only such mHealth efforts are designed for implementation and evaluation [21].

The next phase of this project will also inform programs and policy changes that can support a sustained model of inclusive leisure activities, mHealth integration into macrosystems of information sharing, and equitable distribution of health-promoting opportunities for children with disabilities and their families.

## Abbreviations

mHealth: mobile health
OT: occupational therapist
PAR: participatory action research
REDCap: Research Electronic Data Capture
